# Microwave-Assisted Synthesis of N, S Co-Doped Carbon Quantum Dots for Fluorescent Sensing of Fe(III) and Hydroquinone in Water and Cell Imaging

**DOI:** 10.3390/nano14221827

**Published:** 2024-11-14

**Authors:** Zhaochuan Yu, Chao Deng, Wenhui Ma, Yuqian Liu, Chao Liu, Tingwei Zhang, Huining Xiao

**Affiliations:** 1 International Innovation Center for Forest Chemicals and Materials and Jiangsu Co-Innovation Center for Efficient Processing and Utilization of Forest Resources, Nanjing Forestry University, Nanjing 210037, China; 2 College of Chemistry and Chemical Engineering, Qiqihar University, Qiqihar 161006, China; 3 Macromolecular Chemistry and Bavarian Polymer Institute, University of Bayreuth, 95440 Bayreuth, Germany; 4 Department of Chemical Engineering, University of New Brunswick, Fredericton, NB E3B 5A3, Canada

**Keywords:** microwave irradiation, co-doped carbon quantum dots, fluorescence sensing, Fe^3+^ and hydroquinone detection, cell imaging

## Abstract

The detection of heavy metal ions and organic pollutants from water sources remains critical challenges due to their detrimental effects on human health and the environment. Herein, a nitrogen and sulfur co-doped carbon quantum dot (NS-CQDs) fluorescent sensor was developed using a microwave-assisted carbonization method for the detection of Fe^3+^ ions and hydroquinone (HQ) in aqueous solutions. NS-CQDs exhibit excellent optical properties, enabling sensitive detection of Fe^3+^ and HQ, with detection limits as low as 3.40 and 0.96 μM. Notably, with the alternating introduction of Fe^3+^ and HQ, NS-CQDs exhibit significant fluorescence (FL) quenching and recovery properties. Based on this property, a reliable “on-off-on” detection mechanism was established, enabling continuous and reversible detection of Fe^3+^ and HQ. Furthermore, the low cytotoxicity of NS-CQDs was confirmed through successful imaging of HeLa cells, indicating their potential for real-time intracellular detection of Fe^3+^ and HQ. This work not only provides a green and rapid synthesis strategy for CQDs but also highlights their versatility as fluorescent probes for environmental monitoring and bioimaging applications.

## 1. Introduction

Heavy metal ions and chemical waste are two typical pollutants in environmental contamination, posing significant threats to aquatic ecosystems and human health [1,2,3]. Among heavy metal ions, Fe(III) plays an important role in cellular metabolism and redox reactions, but excess amounts may lead to cellular damage and various diseases [4,5,6,7,8]. Hydroquinone (HQ) is an important chemical raw material and antioxidant, but it has highly biological toxicity and can be absorbed through the skin [9,10]. Long-term exposure may cause skin lesions, liver and kidney damage, and even have carcinogenic potential [11,12] Therefore, the effective detection of Fe(III) and HQ in water is crucial for water quality monitoring and environmental management.

Currently, traditional methods for detecting Fe(III) and HQ include atomic absorption spectroscopy [13], spectrophotometry [14,15], and electrochemical analysis [16,17,18]. These methods can achieve high accuracy and low detection limits under laboratory conditions, but due to their complex operation, expensive equipment, and long detection times, they are challenging to apply for rapid on-site detection [19]. In contrast, fluorescence (FL) methods, with their simplicity of operation, rapid response, and high sensitivity, have increasingly been used for detecting environmental pollutants [20,21]. Yu et al. successfully constructed two three-dimensional porous structures (Eu-MOF and Tb-MOF) for the rapid and sensitive detection of Fe^3+^ in water by anchoring Eu^3+^/Tb^3+^ and rigid 1,2,4,5-benzenedicarboxylic acid imidazole units to their framework [22]. Lan et al. developed a novel bifunctional oligo-thiophene-based fluorescent sensor, which was successfully used for the highly selective and sensitive detection of Fe^3+^ and Hg^2+^ in aqueous solutions [23]. Li et al. prepared a water-soluble molybdenum disulfide quantum dot that can detect HQ with high sensitivity and selectivity [24]. However, traditional fluorescent probe materials such as metal nanoparticles and organic dyes exhibit poor biocompatibility and environmental stability, which may affect biological samples [25]. Therefore, finding a safe and efficient fluorescent probe material has become a research focus.

Carbon quantum dots (CQDs), as a novel fluorescent nanomaterial, have gradually become an ideal candidate in the field of FL sensing due to their unique optical properties, good biocompatibility, and environmental friendliness [26]. Compared to traditional fluorescent materials, CQDs are not only simple to synthesize but also exhibit higher stability and photostability in aqueous solutions [27,28], making them significantly advantageous in the detection of Fe^3+^ and HQ. Studies have shown that doping CQDs with heteroatoms, such as N, S, P, B, and F, can effectively tune their optical properties and surface chemistry, improve the electronic structure of CQDs, increase fluorescence quantum yield, and enhance interactions with target substances, thereby improving detection sensitivity and selectivity [29,30]. Among the current CQD preparation methods, hydrothermal, solvothermal, and microwave-assisted carbonization methods have gained wide attention due to their simplicity and environmental friendliness [27,31]. Yu et al. synthesized nitrogen-doped carbon quantum dots with high quantum yield in a one-step hydrothermal method, enabling sensitive and selective detection of Fe^3+^ [32]. Jaiswal et al. prepared N and S co-doped carbon quantum dots using hydrothermal methods, serving as highly sensitive fluorescent sensors for HQ [33]. In addition to the commonly used hydrothermal method, microwave-assisted synthesis significantly shortens preparation time and increases the yield of CQDs by rapidly heating the reaction system in a short duration [34,35]. Additionally, this method exhibits good adaptability to various precursors and reaction conditions, making it clearly advantageous for preparing high-performance CQDs [36,37].

**Scheme 1:**
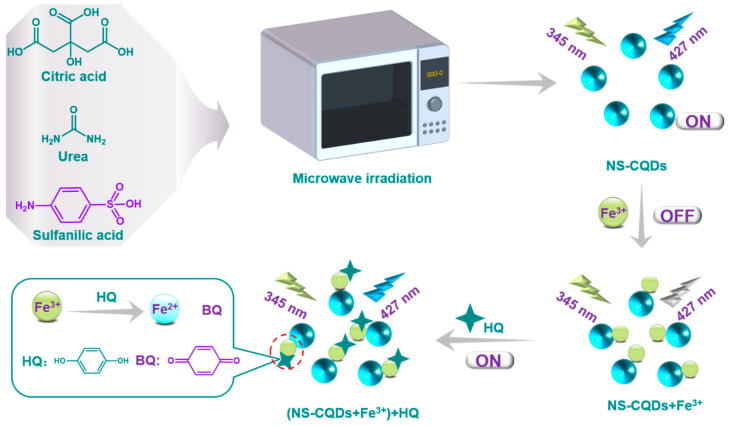
Schematic of the fabrication of co-doped NS-CQDs and its applications.

Herein, we developed a unique strategy for the one-step synthesis of nitrogen and sulfur co-doped carbon quantum dots (NS-CQDs) using a microwave-assisted carbonization method. Using citric acid as the carbon source and urea and *p*-aminobenzenesulfonic acid as nitrogen and sulfur sources, the synthesized NS-CQDs exhibited excellent optical properties in an aqueous solution, enabling efficient, rapid, and sensitive detection of Fe^3+^ and HQ (Scheme 1). Notably, the fluorescence intensity of NS-CQDs rapidly quenched upon the introduction of Fe^3+^, and quickly recovered after the addition of HQ. Based on this rapid FL quenching and recovery effect, we established an “on-off-on” detection mechanism that enables alternating and continuous recognition of Fe^3+^ and HQ, with detection limits as low as 3.40 and 0.96 μM, respectively. Additionally, this sensor demonstrated excellent detection performance in real water systems and was successfully applied to HeLa cell imaging, achieving real-time monitoring of intracellular Fe^3+^ and HQ levels. The establishment of the “on-off-on” fluorescence detection mechanism not only provides a new solution for the detection of Fe^3+^ and HQ in water, but also expands the application perspectives of carbon quantum dots in the field of biological detection.

## 2. Experimental Section

### 2.1. Materials

Citric acid (CA, 98.0%), 4-anilinesulfonic acid (99.0%), hydroquinone (HQ, ≥99%), resorcinol (99%), phloroglucinol (99.0%), L-lysine (L-lys, 98%), and L-cysteine (L-cyc, 99%) were purchased by J&K Scientific Ltd., Beijing, China. Urea (99.8%), p-aminophenol (≥99%), and m-aminophenol (98%) were provided by Tianjin Komiou Chemical Reagent Co., Ltd., Tianjing, China. Phenol (99%) and glucose (99%) were purchased from Beijing Chengyu Chemical Co., Ltd., Beijing, China. The cations used for detection were all nitrate cations purchased from Nanjing Chemical Co., Ltd., Nanjing, China. All the solvents used in the experiments were analytical grade and used without further purification.

### 2.2. Preparation of N and S Co-Dopped Carbon Quantum Dots (NS-GQDs)

N and S co-doped carbon quantum dots (NS-CQDs) were prepared via a microwave-assisted carbonization method [38]. First, citric acid (210 mg, 1 mmol), urea (180 mg, 3 mmol), and 4-aminobenzenesulfonic acid (85 mg, 0.5 mmol) were dissolved in 5 mL of distilled water and sonicated for 30 min to form a uniformly dispersed solution. Then, the mixed solution was transferred to a 25-mL crucible and subjected to microwave heating at 800 W for varying durations (3, 4, and 5 min). The resulting product solution was dialyzed in a dialysis bag (MW cutoff: 1000 Da) for 72 h, with the retained solution collected. Subsequently, the retained solution was placed in a dialysis bag with a molecular weight cutoff of 110,000 Da and dialyzed for an additional 48 h to remove larger particles. Finally, the dialysate was collected and freeze-dried to obtain 90 mg of yellow sponge-like NS-CQDs.

### 2.3. Fluorescence Measurements

In the fluorescence (FL) detection process of Fe^3+^ and hydroquinone (HQ), 30 mg of NS-CQDs were dispersed in 1 L of deionized (DI) water and sonicated for 30 min (100 W) to obtain a uniformly dispersed NS-CQDs solution. Subsequently, different concentrations of Fe^3+^ solution were added to the system, and after shaking for 30 min, the FL intensity of the system was measured (the initial FL intensity was denoted as F_0_). The sensitivity and dynamic detection range of the NS-CQDs sensor were evaluated through the concentration-dependent FL ratio plot of Fe^3+^-NS-CQDs dispersion. To assess the effect of other competing ions on the Fe^3+^-NS-CQDs FL sensing, interference experiments were conducted, selecting ions (K^+^, Ca^2+^, Na^+^, Mg^2+^, Zn^2+^, Co^2+^, Ni^2+^, Cu^2+^, Hg^2+^, Ag^+^, Pb^2+^, Cd^2+^, and Cr^3+^) at three times the concentration of Fe^3+^. Additionally, to test the detection sensitivity of the NS-CQDs+Fe^3+^ system for HQ and its potential interfering substances (such as citric acid, resorcinol, phloroglucinol, L-lysine, L-cysteine, p-aminophenol, m-aminophenol, and phenol), competitive experiments were conducted using the same method as for Fe^3+^ detection. Three parallel experiments were conducted to ensure data reliability. The quantum yield (QY) of NS-CQDs was measured according to reported methods. A standard solution of quinine sulfate (QY = 0.54) was dissolved in 0.1 M H_2_SO4, and the fluorescence emission spectra and UV absorption spectra of both the standard and NS-CQDs will be measured. The relative quantum yield of NS-CQDs can be calculated using the following formula (1):

(1) $$\Phi_{sample}=\Phi_{std}\times (\frac{I_{sample}}{I_{std}})\times (\frac{{Abs}_{std}}{{Abs}_{sample}})\times (\frac{\eta_{sample}}{\eta_{std}}{)}^{2}$$

Φ_sample_, Φ_std_, I_sample_, I_std_, Abs_sample_, Abs_std_, η_sample_, and η_std_ represent the quantum yield, FL emission intensity, absorption at the excitation wavelength, and refractive index for the sample NS-CQDs and the standard dye, respectively.

### 2.4. Detection of Fe^3+^ and HQ in Actual Water Samples

Actual water samples were collected from lake water (Labor Lake in Qiqihar (Qiqihar, China)), river water (the Nenjiang River (Nenjiang, China)), and tap water (Qiqihar, China). The samples were filtered through a 0.22 μm microporous membrane and then diluted 100 times before use. The water samples were added to NS-CQDs, and the FL intensity was measured, followed by spike recovery experiments. All experiments were conducted at room temperature and repeated three times.

### 2.5. Cytotoxicity and Imaging Studies

To evaluate the biocompatibility of NS-CQDs, we incubated HeLa cells with different concentrations of NS-CQDs (12.5–200 mg L^−1^) and assessed cytotoxicity using the MTT assay. Briefly, the cells were seeded at a density of 1 × 10^4^ cells/well in a 96-well plate using culture medium containing 10% fetal bovine serum (FBS) and incubated for 24 h at 37 °C with 5% CO_2_. Subsequently, 12.5–200 mg L^−1^ of NS-CQDs was added, and the cells were further incubated at 37 °C for 24 h. After incubation, the culture medium was discarded, and the cells were washed three times with PBS buffer. Then, 0.5 mg mL^−1^ of MTT solution was added to each well and incubated for 3–4 h, followed by the addition of 0.1 mL DMSO to dissolve the formazan crystals formed. The absorbance (OD value) was measured at 570 nm using a SpectraMax M2e microplate reader, and cell viability was calculated based on the OD values. The cell survival rate was calculated as follows:

$$Cellviability\left(\%\right)=\frac{{OD}_{s}-{OD}_{b}}{{OD}_{c}-{OD}_{b}}\times 100\%$$

where OD_s_, OD_b_, and OD_c_ are the OD values at 570 nm for the experimental group, blank group, and control group, respectively.

For cell imaging, the cultured HeLa cells were incubated with NS-GQDs at a concentration of 30 mg L^−1^ for 4 h, followed by incubation in mixed phosphate buffer for 4 h. Subsequently, Fe^3+^ (300 μM) and HQ (300 μM) were added sequentially, with each incubation lasting 4 h. Finally, confocal microscope images under three different conditions were observed and recorded using laser scanning confocal microscopy.

### 2.6. Characterization

The morphologies of individual NS-CQDs were observed using a high-transmission electron microscope (TEM, Tecnai G2 F30, Hillsboro, OR, USA). An atomic force microscopy (AFM) (Multomode8, Veeco Instruments Inc., Tucson, AZ, USA) was used to examine the morphology of the NS-CQDs sample. X-ray diffractometer was used to observe X-ray diffraction spectra of the materials (D8-FOCUS, K α Rigaku, 10° min^−1^, Bruker Corporation, BW, Karlsruhe, Germany). X-ray photoelectron spectroscopy (XPS, ESCALAB 250Xi, Thermo Fisher Inc., Waltham, MA, USA) and a Spectrum One spectrometer (FT-IR Perkin Elmer, Hopkinton, MA, USA) were used to analyze the chemical composition and state of all prepared samples. The absorbance, the Raman spectroscopy, and the fluorescence spectrum of the samples were collected using a UV-Vis spectrophotometer (TU-1901, Shimadzu, Tokyo, Japan), HJYlabRAMHR-Evdotion UV confocal Raman spectrometer, the FLS920 Transient/Steady-State Fluorescence Spectrophotometer (Edinburgh Instruments Ltd., Livingston, UK), and the FL55 fluorescence spectrophotometer (Perkin Elmer, Hopkinton, MA, USA). An IX51 inverted fluorescence microscope (Olympus Corporation, Tokyo, Japan) was used for imaging HeLa cells.

## 3. Results and Discussion

### 3.1. Synthesis and Characterization of NS-CQDs

**Figure 1:**
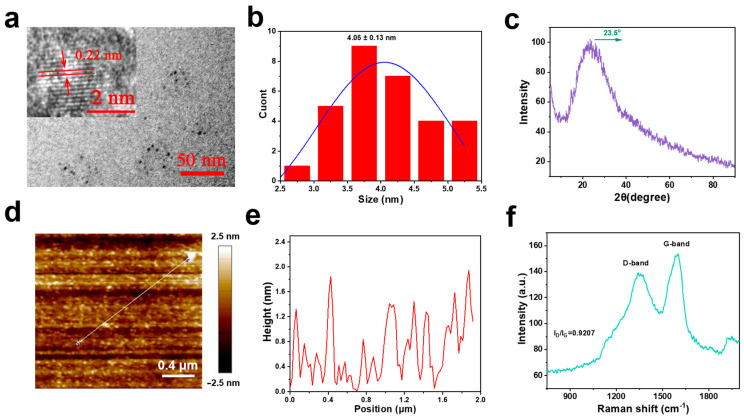
Synthesis and characterization of NS-CQDs. (**a**) HRTEM image of NS-CQDs (the inset shows its lattice fringes). (**b**) Diameter distribution analysis, (**c**) XRD, (**d**) AFM image, (**e**) height distribution analysis, and (**f**) Raman spectroscopy of NS-CQDs.

Numerous studies have reported methods for preparing carbon dots, particularly the use of urea and citric acid as nitrogen and carbon sources [36,37,38]. In this study, a simple and efficient microwave-assisted pyrolysis method was used to prepare sulfur and nitrogen co-doped carbon quantum dots (NS-CQDs). Specifically, urea, citric acid, and p-aminobenzenesulfonic acid were used as precursors dissolved in deionized water and microwaved at 800 W for varying durations. By adjusting the microwave time, the size and distribution of the NS-CQDs were optimized. When the microwave heating time was 4 min (Figure 1a), the NS-CQDs had the smallest and most uniform size distribution, with an average diameter of approximately 4.1 nm (Figure 1b). Beyond 6 min, the precursors were almost completely carbonized (Figure S1), indicating that reaction time directly affects the degree of carbonization. TEM analysis showed that the NS-CQDs have a lattice spacing of 0.22 nm, corresponding to the (110) plane of graphene (Figure 1a inset) [39]. XRD data further supported this conclusion, showing a broad diffraction peak at 2θ = 23.5°, attributed to the (002) plane of graphite (Figure 1c) [40]. Additionally, Raman spectra showed D and G peaks at 1340 cm^−1^ and 1580 cm^−1^, respectively (Figure 1d). The intensity ratio of the D and G peaks (I_D_/I_G_) was 0.9207, indicating significant defects in the material, consistent with heteroatom doping and the presence of oxygen-containing functional groups [41]. Moreover, AFM measurements showed that the thickness of NS-CQDs was less than 2.0 nm (Figure 1e,f), further confirming their graphene-like crystal structure and substantial structural defects. These results indicate that, by controlling appropriate reaction conditions, uniform-sized and structurally stable NS-CQDs were successfully prepared.

**Figure 2:**
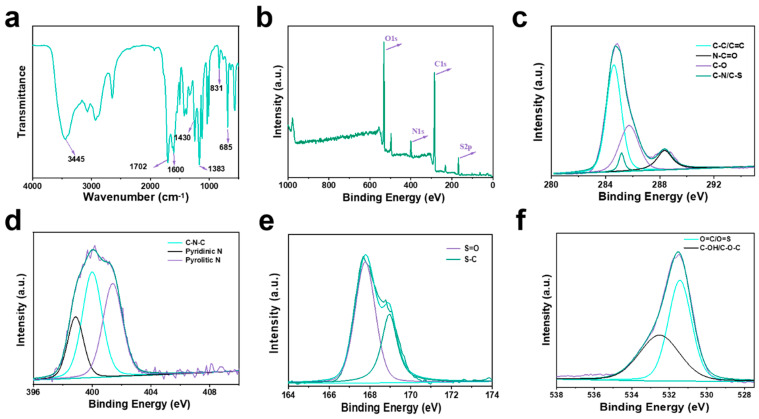
Chemical composition analysis of NS-CQDs. (**a**) FTIR and (**b**) XPS spectra of NS-CQDs. (**c**–**f**) High-resolution XPS of C 1s, N 1s, S 2p, and O 1s, respectively.

Subsequently, the structure and surface chemical composition of NS-CQDs were characterized by FT-IR and XPS spectroscopy. In the FT-IR spectrum, a distinct absorption peak was observed at 1600 cm^−1^, corresponding to the skeletal vibrations of graphene, indicating the formation of a significant amount of sp^2^ carbon structures during the preparation, which share similar structural characteristics with graphene (Figure 2a) [42]. Additionally, the absorption peaks at 1633 and 1702 cm^−1^ were attributed to surface C=O groups, while the broad peaks at 3445 and 1383 cm^−1^ corresponded to the stretching and bending vibrations of -OH, respectively [41]. The strong absorption peaks at 1036 and 1431 cm^−1^ were attributed to the O=S=O bonds of -SO_3_H and the stretching vibrations of C-N, respectively [43]. The FT-IR results indicated successful doping of N and S elements, and the NS-CQDs were rich in various oxygen-containing functional groups, such as -COOH, -OH, and -SO_3_H. As shown in Figure 2b, the XPS survey spectrum of NS-CQDs displayed distinct signals of S, C, N, and O elements at 167.1, 284.6, 400.8, and 532.3 eV, respectively [44]. The high-resolution C1s spectrum showed peaks at 284.6, 285.2, 285.7, and 288.4 eV, corresponding to C-C/C=C, C-N/C-S, C-O, and N-C=O bonds, respectively (Figure 2c) [45]. The high-resolution N1s spectrum displayed peaks at 398.9, 400.0, and 401.4 eV, corresponding to C-N-C bonds, pyridinic nitrogen, and pyrrolic nitrogen, respectively (Figure 2d) [46]. The two peaks in the S2p spectrum at 167.7 and 168.9 eV were attributed to S=O and S-C bonds (Figure 2e) [47,48]. The two peaks at 531.5 and 532.6 eV in the O1s spectrum are attributed to O=C/O=S and C-OH/C-O-C bonds (Figure 2f) [46]. These results were consistent with the FT-IR analysis, further confirming the successful doping of N and S elements and indicating that the prepared NS-CQDs possess good structural characteristics and rich surface functional groups.

### 3.2. Photoluminescence Study of NS-CQDs

**Figure 3:**
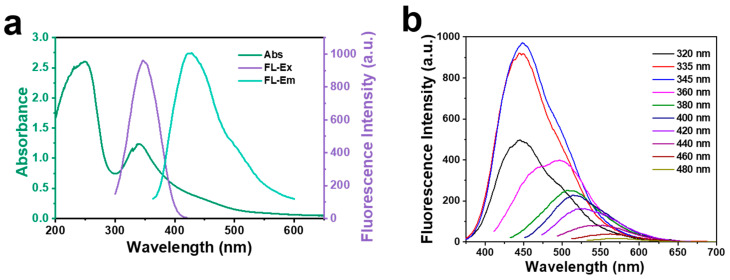
Evaluation of FL properties of NS-MQDs. (**a**) UV-vis absorption spectra and FL excitation (Ex) and FL emission (Em) spectra of NS-MQDs in aqueous solution. (**b**) FL emission spectra of NS-MQDs at different excitation wavelengths (320–480 nm).

The optical properties of NS-CQDs are particularly crucial when analyzing their applications. As shown in Figure 3a, the absorption spectrum of NS-CQDs exhibits a strong absorption band in the 200–300 nm range and another peak near 345 nm, corresponding to π-π* transitions and n-π* transitions of the NS-CQDs, respectively. The excitation (Ex) spectrum coincides closely with the n-π* transition band, with a peak at 345 nm, indicating that n-π* transitions are the primary source of the fluorescence emission (Em) NS-CQDs [49]. As shown in Figure 3b, the Em behavior of NS-CQDs exhibits typical excitation-dependent characteristics, reflecting the complex luminescence mechanism of NS-CQDs [50]. Moreover, when the excitation wavelength is 345 nm, the corresponding maximum Em wavelength is 427 nm, which is consistent with the analysis results of the UV-visible absorption spectrum, further confirming the fluorescence Em mechanism of NS-CQDs. This is consistent with the ultraviolet absorption and FL emission characteristics reported for carbon dots prepared by the pyrolysis of citric acid and urea [50,51,52,53]. Notably, the quantum yield (QY) of carbon quantum dots (CQDs) typically prepared by the pyrolysis of citric acid and urea ranges from 2% to 15% [52,54,55], while the N, S co-doped NS-CQDs achieve a slightly higher QY of 17.1%. This improvement in QY may be attributed to the doping effect of heteroatoms: on one hand, it modulates the energy band structure of the quantum dots, and on the other, it passivates surface defects, enhances charge carrier separation efficiency, and introduces new luminescent centers, significantly boosting fluorescence efficiency [56,57,58]. Furthermore, the QY of NS-CQDs is similar to that of microwave-synthesized carbon quantum dots reported in the literature (Table S1), offering potential for applications in bioimaging, optical sensing, and environmental monitoring.

### 3.3. NS-CQDs Sensor for Detection of Fe^3+^ and HQ

**Figure 4:**
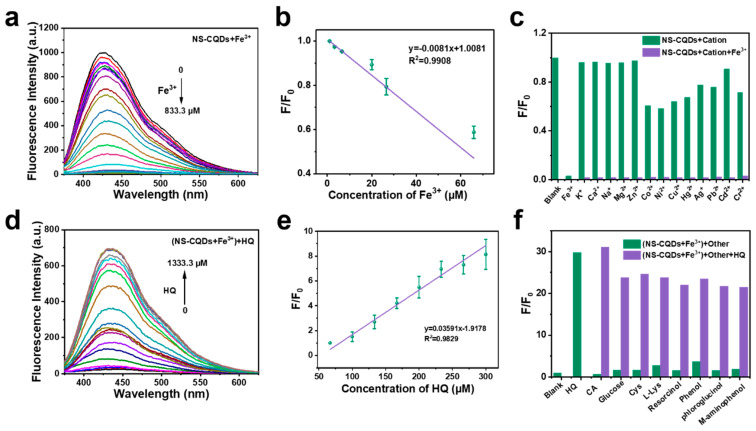
“On-off-on” detection performance of NS-CQDs for Fe^3+^ and HQ. (**a**) The concentration-dependent emission spectra of NS-CQDs at different concentrations of Fe^3+^ (0–833.3 μM). (**b**) Stern-Volmer plot of F/F_0_ versus Fe^3+^ concentration for NS-CQDs. (**c**) FL intensity change profiles of NS-CQDs in the presence of potential competing ions (green) and Fe^3+^ upon addition of competing anions (3-fold excess, purple) in DI water. (**d**) The concentration-dependent emission spectra of (NS-CQDs+Fe^3+^) for different concentrations of HQ (0–1333.3 μM). (**e**) Stern-Volmer plot of F/F_0_ versus HQ concentration for (NS-CQDs+Fe^3+^). (**f**) FL intensity change profiles of NS-CQDs in the presence of potential competing ions (green) and HQ upon addition of competing anions (3-fold excess, purple) in DI water.

Carbon quantum dots (CQDs) are widely used in the sensing and detection of heavy metal ions due to their excellent optical and photoluminescent (FL) properties [28,59]. To this end, a systematic study was conducted to evaluate the heavy metal ion detection capability of NS-CQDs. Selectivity, a key indicator of sensor performance, was assessed using a range of metal ions including K^+^, Ca^2+^, Na^+^, Mg^2+^, Zn^2+^, Fe^3+^, Co^2+^, Ni^2+^, Cu^2+^, Hg^2+^, Ag^+^, Pb^2+^, Cd^2+^, and Cr^3+^ (Ex: 345 nm) to evaluate the selectivity of NS-CQDs. The results show that although various cations caused different degrees of FL quenching, Fe^3+^ exhibited the most significant quenching effect, with a quenching efficiency of up to 97.1% (Figure S2). The FL titration spectra of NS-CQDs for Fe^3+^ are shown in Figure 4a and Figure S3, where the FL intensity gradually decreases as the Fe³⁺ concentration increases from 0 to 833.3 μM. When the Fe^3+^ concentration reached 300 μM, the quenching efficiency reached its maximum and stabilized. Furthermore, Figure 4b shows a good linear relationship for Fe^3+^ in the concentration range of 1–66 μM, with a limit of detection (LOD) calculated as 3.4 μM based on 3σ/K (R^2^ = 0.9908). The competitive experiment of NS-GQDs for Fe^3+^ (Figure 4c) showed that the presence of other metal ions (K^+^, Ca^2+^, Na^+^, Mg^2+^, Zn^2+^, Co^2+^, Ni^2+^, Cu^2+^, Hg^2+^, Ag^+^, Pb^2+^, Cd^2+^, and Cr^3+^ at 1000 μM) did not significantly affect the FL response of NS-GQDs to Fe^3+^. Response time testing results (Figure S4) showed that after the addition of Fe^3+^, the FL intensity of the system rapidly decreased within 1 min and remained stable. Therefore, NS-GQDs not only exhibit excellent selectivity and anti-interference capabilities but also provide rapid and sensitive detection of Fe^3+^.

Another significant finding is that the NS-CQDs+Fe^3+^ system can be used to detect hydroquinone (HQ), due to the strong interaction between Fe^3+^ and HQ [53]. When HQ (0–1333 μM) was mixed with the NS-CQDs+Fe^3+^ system, the FL intensity at 427 nm gradually increased, as shown in Figure 4d and Figure S5, indicating that an HQ sensor can be constructed based on the NS-CQDs+Fe^3+^ system. As shown in Figure 4e, HQ exhibits a good linear relationship from 66.7 to 300 μM. The FL intensity ratio was fitted to a linear equation with HQ concentration, yielding a limit of detection (LOD) of 0.96 μM (R^2^ = 0.9829). Compared to most reported Fe^3+^ and HQ detection materials (Table S2), the NS-CQDs have a lower detection limit, showing potential advantages in high-sensitivity detection. Furthermore, compared to other potential competitors (CA, glucose, Cys, L-Lys, resorcinol, phenol, phloroglucinol, and m-aminophenol) for HQ, only HQ could restore the fluorescence intensity at 427 nm (Figure 4f), demonstrating the high selectivity of the HQ sensor. As shown in Figure S6, when 633.3 μM HQ was added to the NS-CQDs+Fe^3+^ system, the FL intensity gradually increased within the first 0–5 min, making the optimal response time 5 min. The wide linear range and low detection limit of the sensor for HQ detection provide favorable conditions for its practical application. Additionally, NS-GQDs exhibit good reversibility in the detection of Fe^3+^ and HQ (Figure S7), allowing for multiple repetitions of the recognition process with minimal loss in fluorescence efficiency. Therefore, NS-GQDs can be utilized to establish an “on-off-on” fluorescence detection strategy for the simultaneous identification of Fe^3+^ and HQ. Although Fe^3+^ and HQ detection materials based on the “on-off-on” mechanism have been reported, most can only detect one of them individually, making it challenging to achieve consecutive detection of both Fe^3+^ and HQ [53]. This further highlights the unique advantage of our material in simultaneous detection.

To verify the application effectiveness of NS-GQDs in real water samples, lake water, river water, and tap water were selected as samples, and the concentrations of Fe^3+^ and HQ were measured using FL spectroscopy combined with the standard addition method. The results, as shown in Tables S3 and S4, indicate that the recoveries of Fe^3+^ and HQ in the three water samples ranged from 98.2% to 103.4%, with relative standard deviations (RSD) of less than 3.48%, demonstrating good accuracy and precision of the detection results. These results confirm that NS-GQDs have good applicability in detecting Fe^3+^ and HQ in real water samples and can effectively eliminate the influence of other potential interfering substances.

### 3.4. Mechanism of Fluorescence “On-Off-On” Detection for Fe^3+^ and HQ

**Figure 5:**
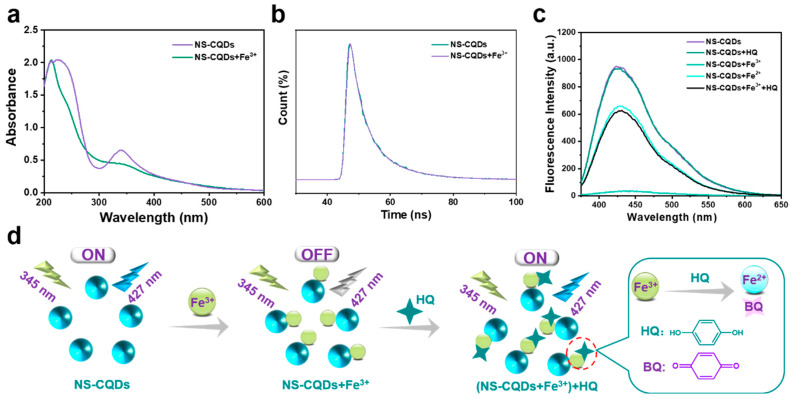
Fluorescence “on-off-on” principle of NS-CQDs. (**a**) UV-vis absorption spectrum and (**b**) FL decay curves of NS-CQDs before and after the addition of Fe^3+^. (**c**) FL spectrum of NS-CQDs, NS-CQDs+HQ, NS-CQDs+Fe^3+^, NS-CQDs Fe^2+^, and NS-CQDs+Fe^3+^+HQ. (**d**) Schematic of the proposed “on-off-on” mechanism for the detection of Fe^3+^ and HQ using NS-CQDs.

To explore the mechanism of the observed FL “on-off-on” effect during testing, FL decay curves and UV-vis absorption spectra were obtained (Figure 5a,b). As shown in Figure 5a, the absorption band of NS-CQDs at 345 nm changed significantly after the introduction of Fe^3+^ ions, indicating a possible interaction between the quantum dots and Fe^3+^. This change may result from the binding of Fe^3+^ to the quantum dots, forming a new complex or altering the electronic environment of CQDs, thereby affecting their optical properties. Although changes occurred in the UV-vis absorption spectra, the FL decay curves of the NS-CQDs system showed no significant changes before and after the addition of Fe^3+^, with FL lifetimes of 7.42 ns and 7.48 ns, respectively (Figure 5b), suggesting a possible static quenching mechanism [40,51]. Based on the calculation using the Stern-Volmer equation, the quenching rate constant was found to be Kq = 7.2 × 10^11^ M^−1^ s^−1^, which exceeds the maximum value for dynamic quenching effects (1.0 × 10^10^ M^−1^ s^−1^), indicating that the cause of FL quenching in NS-GQDs may be static quenching, suppressing the fluorescence emission of the quantum dots [53,60,61]. This suggests that the interaction between Fe^3+^ and the quantum dots primarily leads to a decrease in FL intensity through binding rather than energy transfer.

To investigate the mechanism of FL recovery after the introduction of HQ, the FL spectra of NS-CQDs were studied following the addition of Fe^3+^, Fe^2+^, and HQ (Figure 5c). When HQ was added first, the FL intensity of NS-CQDs remained unchanged, but it significantly decreased after the addition of Fe^3+^. Notably, the FL intensity of the system with Fe^3+^ and HQ added sequentially was similar to that observed after the direct addition of Fe^2+^. This indicates that in the NS-CQDs+Fe^3+^ and HQ system, Fe^3+^ may oxidize HQ to quinone while reducing itself to Fe^2+^, leading to the recovery of the fluorescence in the system [51]. The quenching effect of Fe^2+^ on the FL of NS-CQDs is relatively minor. Therefore, the proposed “on-off-on” FL mechanism for NS-CQDs is illustrated in Figure 5d.

### 3.5. Fluorescence Imaging of NS-CQDs in Living Cells

**Figure 6:**
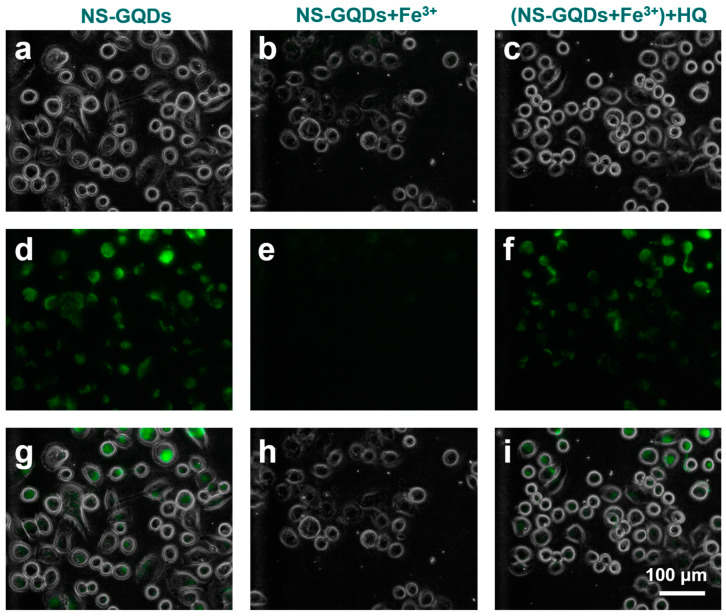
Confocal FL images of living HeLa cells. The images after incubating NS-CQDs at 37 °C for 4 h are as follows: (**a**) bright field, (**d**) FL, and (**g**) merged image. The images of HeLa cells stained with NS-CQDs after treatment with Fe^3+^ (300 μM) for 4 h are: (**b**) bright field, (**e**) FL, and (**h**) merged image. The images of HeLa cells stained with NS-CQDs after treatment with Fe^3+^ (300 μM) followed by HQ (300 μM) for 4 h are: (**c**) bright field, (**f**) FL, and (**i**) merged image.

Imaging Fe^3+^ and HQ in cells is crucial for understanding their roles in redox processes and oxidative stress, which are involved in various physiological and pathological mechanisms [62,63,64]. NS-CQDs exhibit good water dispersibility and excellent optical properties, making them well-suited for imaging Fe^3+^ and HQ in cells. Biocompatibility is crucial for their potential in cell imaging and clinical applications. In cytotoxicity tests, HeLa cells coincubated with different concentrations of NS-CQDs (12.5–200 mg L^−1^) for 24 h showed a cell viability of over 91.8 ± 0.9% (Figure S8), indicating low toxicity and suitability for monitoring Fe^3+^ and HQ in complex biological environments. The imaging performance of NS-CQDs within cells was observed using confocal laser scanning microscopy (CLSM). The results showed that HeLa cells treated with NS-CQDs for 4 h exhibited bright green fluorescence under 345 nm excitation, with clear cell morphology and contours, indicating the ability of NS-CQDs to penetrate the cell membrane (Figure 6). To investigate the detection of Fe^3+^ and HQ by NS-CQDs within cells, Fe^3+^ and HQ were sequentially introduced into the treated HeLa cells. Upon introducing Fe^3+^, the intracellular fluorescence was significantly quenched, and the fluorescence was restored upon adding HQ, confirming the potential of NS-CQDs for detecting and real-time monitoring of Fe^3+^ and HQ within cells. This “on-off-on” sensing capability offers significant promise for environmental monitoring and early disease diagnosis, highlighting the potential of NS-CQDs in biomedical imaging applications.

## 4. Conclusions

In this study, we synthesized nitrogen and sulfur co-doped carbon quantum dots (NS-CQDs) through a microwave-assisted carbonization method using citric acid, urea, and p-aminobenzenesulfonic acid as precursors. The NS-CQDs exhibited excellent optical properties, enabling their use as an efficient fluorescent sensor for Fe^3+^ and hydroquinone (HQ) detection in water. The fluorescence of NS-CQDs was significantly quenched by Fe^3+^ and effectively restored upon HQ addition, establishing a reliable “on-off-on” detection mechanism. This sensor achieved low detection limits of 3.40 μM for Fe^3+^ and 0.96 μM for HQ, along with strong selectivity, making it suitable for water quality monitoring. Furthermore, the NS-CQDs demonstrated low cytotoxicity, as confirmed by successful HeLa cell imaging for real-time intracellular Fe^3+^ and HQ detection. This work offers a green and rapid synthesis strategy for CQDs and highlights their potential as versatile fluorescent probes for environmental monitoring and bioimaging applications.

## Data Availability

Data are contained within the article and Supplementary Materials.

## Supplementary Materials

The following supporting information can be downloaded at: https://www.mdpi.com/article/10.3390/nano14221827/s1, Figure S1. HRTEM images of NS-CQDs heated by microwave for different times ((a) 5 min and (b) 6 min). Figure S2. FL spectra of the system after adding different cations (K^+^, Ca^2+^, Na^+^, Mg^2+^, Zn^2+^, Fe^3+^, Co^2+^, Ni^2+^, Cu^2+^, Hg^2+^, Ag^+^, Pb^2+^, Cd^2+^, and Cr^3+^ at 300 μM) to the NS-CQDs dispersion (30 mg L^−1^). Figure S3. FL intensity of NS-CQDs treated with different concentrations of Fe^3+^ (0–833.3 μM). Figure S4. Fluorescent changes of NS-MQDs+Fe^3+^ system with different time (0–10 min). Figure S5. FL intensity of NS-CQDs treated with different concentrations of HQ (0–1333.3 μM). Figure S6. The FL changes of NS-CQDs+Fe^3+^+HQ system with different time (0–30 min). Figure S7. The changes in FL intensity (Em: 427 nm) of the NS-CQDs dispersion upon the alternate introduction of Fe^3+^ and HQ. Figure S8. The cell viability of HeLa cells cultured for 24 h in different concentrations of NS-CQDs (12.5–200 mg L^−1^). Table S1. Comparison of the quantum yield (QY) of NS-CODs with other reported carbon quantum dots. Table S2. Comparison of the detection performance of NS-CQDs for Fe^3+^ and HQ with other reported materials. Table S3. Detection of Fe^3+^ in actual water samples using NS-CQDs. Table S4. Detection of HQ in actual water samples using NS-CQDs+Fe^3+^. References [65,66,67,68,69,70,71,72,73,74,75,76,77,78,79,80] were cited in the Supplementary Materials.
