# Supplementary material for: Microwave-Assisted Synthesis of N, S Co-Doped Carbon Quantum Dots for Fluorescent Sensing of Fe(III) and Hydroquinone in Water and Cell Imaging
Source: Nanomaterials (Basel). 2024 Nov 14;14(22):1827. doi: 10.3390/nano14221827 (PMC11597194; doi:10.3390/nano14221827)
Supplement: Supplementary file 1 [file nanomaterials-14-01827-s001.zip › nanomaterials-3295715-supplementary.pdf]

**Supporting Information**

# Microwave-Assisted Synthesis of N, S Co-Doped Carbon Quantum Dots for Fluorescent Sensing of Fe(III) and Hydroquinone in Water and Cell Imaging

**Zhaochuan Yu [1,2], Chao Deng [1,3,*], Wenhui Ma [2,*], Yuqian Liu [1], Chao Liu [1], Tingwei Zhang [1,*] and Huining Xiao [4]**

[1] International Innovation Center for Forest Chemicals and Materials and Jiangsu Co-Innovation Center for Efficient Processing and Utilization of Forest Resources, Nanjing Forestry University, Nanjing 210037, China; zcyu0818@njfu.edu.cn (Z.Y.); liuyq@njfu.edu.cn (Y.L.); chaoliulc@njfu.edu.cn (C.L.) chao.deng@uni-bayreuth.de (C.D.)
[2] College of Chemistry and Chemical Engineering, Qiqihar University, Qiqihar 161006, China; zcyu0818@njfu.edu.cn (Z.Y.)
[3] Macromolecular Chemistry and Bavarian Polymer Institute, University of Bayreuth, 95440 Bayreuth, Germany
[4] Department of Chemical Engineering, University of New Brunswick, Fredericton, NB E3B 5A3, Canada; hxiao@unb.ca
* Correspondence: chao.deng@uni-bayreuth.de (C.D.); mwh972@qqhru.edu.cn (W.M.); ztwei@njfu.edu.cn (T.Z.)

## Supporting Figures

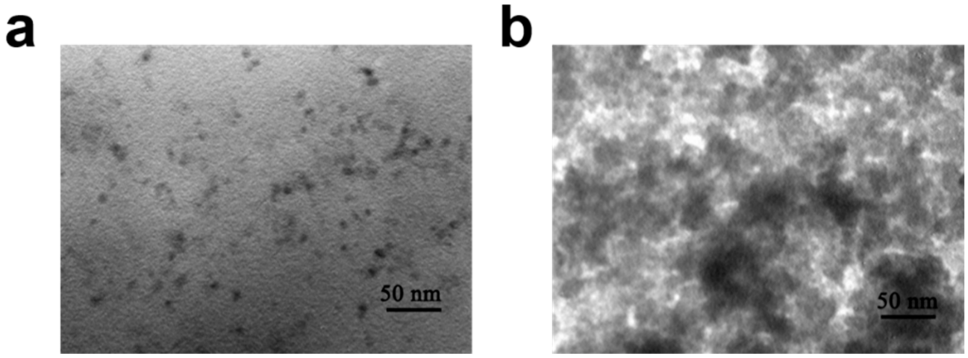


**Fig. S1** HRTEM images of NS-CQDs heated by microwave for different times ((a) 5 min and (b) 6 min).

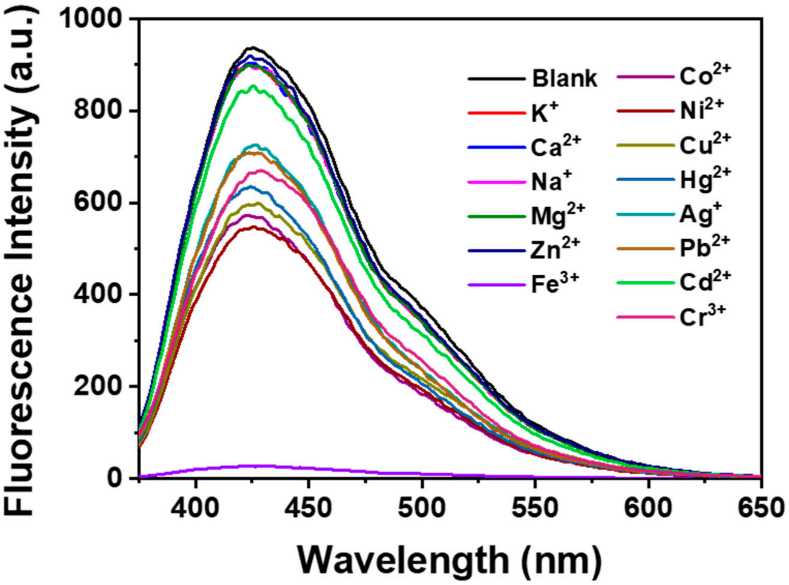


**Fig. S2** FL spectra of the system after adding different cations (($K^+$, $Ca^{2+}$, $Na^+$, $Mg^{2+}$, $Zn^{2+}$, $Fe^{3+}$, $Co^{2+}$, $Ni^{2+}$, $Cu^{2+}$, $Hg^{2+}$, $Ag^+$, $Pb^{2+}$, $Cd^{2+}$, and $Cr^{3+}$ at 300 μM)) to the NS-CQDs dispersion (30 mg $L^{-1}$)

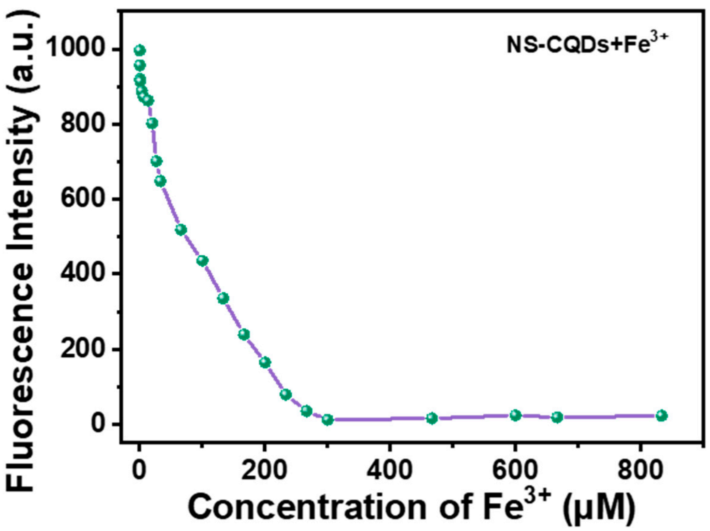

**Fig. S3** FL intensity of NS-CQDs treated with different concentrations of $Fe^{3+}$ (0-833.3 μM)

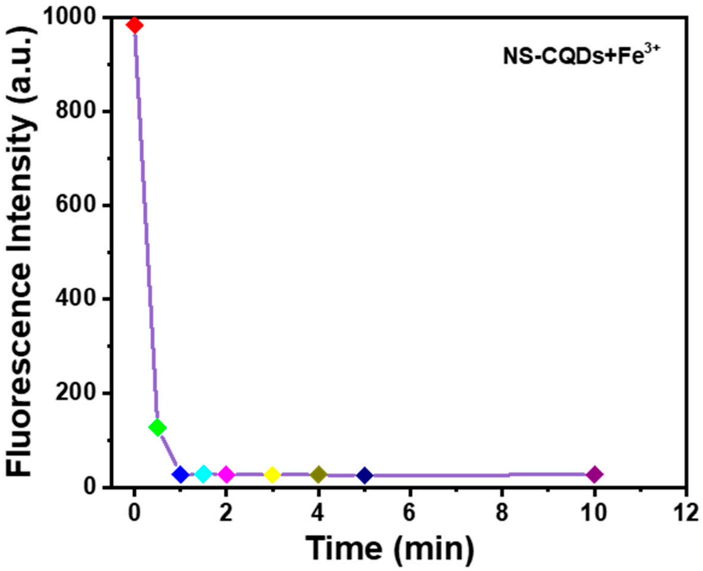


**Fig. S4** Fluorescent changes of NS-MQDs+$Fe^{3+}$ system with different time (0-10 min).

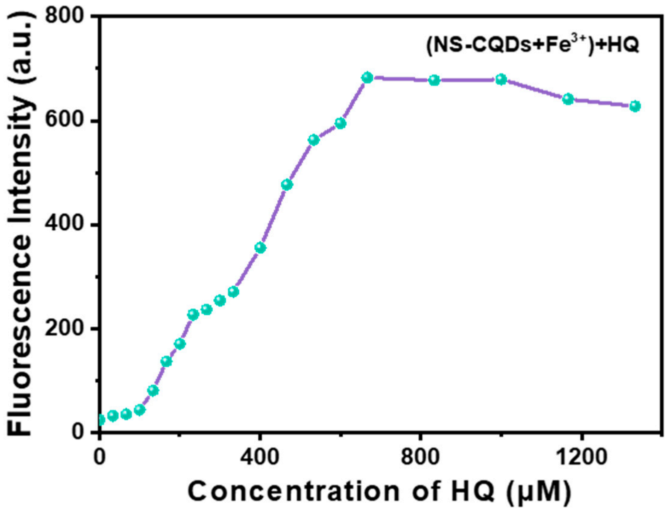


**Fig. S5** FL intensity of NS-CQDs treated with different concentrations of HQ (0-1333.3 μM).

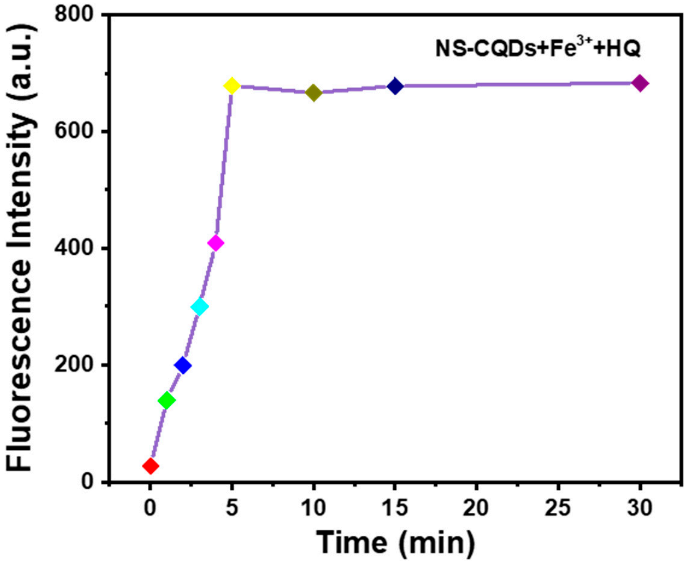


**Fig. S6** The FL changes of NS-CQDs+$Fe^{3+}$+HQ system with different time (0-30 min).

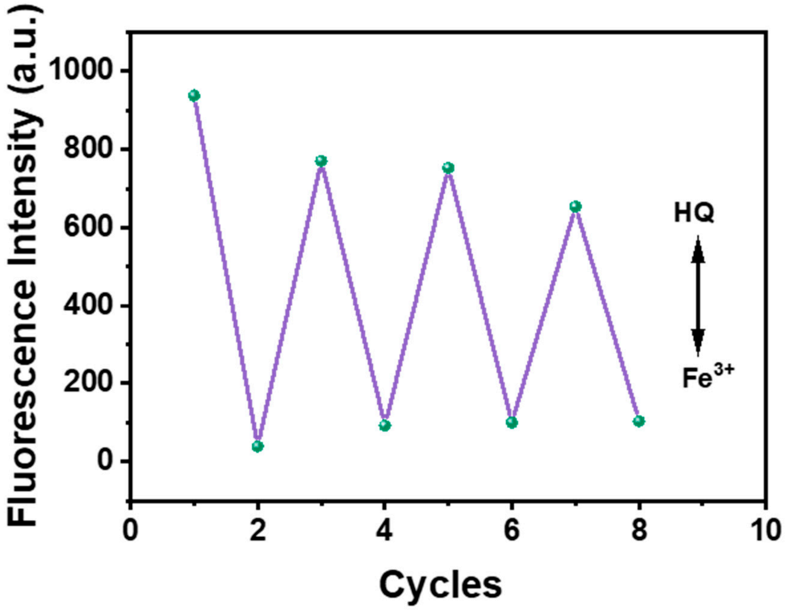


**Fig. S7** The changes in FL intensity (Em: 427 nm) of the NS-CQDs dispersion upon the alternate introduction of $Fe^{3+}$ and HQ.

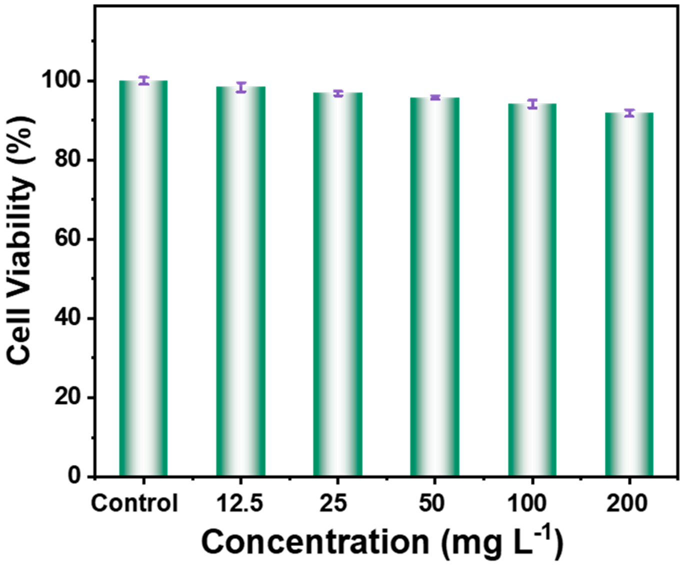


**Fig. S8** The cell viability of HeLa cells cultured for 24 h in different concentrations of NS-CQDs (12.5-200 mg $L^{-1}$).

# Supporting Table

**Table S1.** Comparison of the quantum yield (QY) of NS-CODs with other reported carbon quantum dots.

| | Materials | Precursor | Microwave irradiation time (min) | Quantum yield | Reference |
|---|---|---|---|---|---|
| 1 | CDs | Ethylene glycol; Citric acid | 5 | 0.8% | [65] |
| 2 | CQDs | Phthalic acid; Triethylenediamine hexahydrate | 1 | 16.1% | [66] |
| 3 | TTO-CDs | Tween-20 | 6 | 14% | [67] |
| 4 | GQDs | Aspartic acid; $NH_4HCO_3$ | 10 | 14% | [68] |
| 5 | CQDs | Citric acid, Urea; Thiourea | 5 | 19.2% | [69] |
| 6 | C-dots | Citric acid; Tetraoctylammonium bromide | 3 | 11% | [70] |
| 7 | S-CDs | Citric acid; *L*-cysteine; Dextrin | 3 | 22% | [71] |
| 8 | N, P-C-dots | *N*-phosphonomethyl aminodiacetic acid; Ethylenediamine | 7 | 17.5% | [72] |
| 9 | NS-CQDs | Urea; Citric acid; *p*-aminobenzenesulfonic acid | 5 | 17.1% | This work |

**Table S2** Comparison of the detection performance of NS-CQDs for $Fe^{3+}$ and HQ with other reported materials.

| | Materials | Detection range of $Fe^{3+}$ (μM) | Detection limit of $Fe^{3+}$ (μM) | Detection range of HQ (μM) | Detection limit of HQ (μM) | Reference |
|---|---|---|---|---|---|---|
| 1 | CPDs | 0-10 | 0.34 | - | - | [73] |
| 2 | R-CDs | 0.08-24 | 0.30 | - | - | [74] |
| 3 | CCDs | 0-50 | 4.5 | - | - | [75] |
| 4 | PPET-CDs | 5-50 | 2.14 | - | - | [76] |
| 5 | CDs | 16-166 | 6.05 | - | - | [77] |
| 6 | CQDs-films | - | - | 8.8-1040 | 9.8 | [78] |
| 7 | N,Si-GQDs | - | - | 5.0-200 | 1.35 | [53] |
| 8 | fluorescent nanozyme (Cu-BDC-$NH_2$) | - | - | 0-10 | 0.997 | [79] |
| 9 | GQDs | - | - | 0.1-50 | 0.1 | [80] |
| 10 | N/S/P-CDs | - | - | 0.56-375 | 0.16 | |
| 11 | NS-CQDs | 1-66 | 3.4 | 66.7-300 | 0.96 | This work |

**Table S3** Detection of $Fe^{3+}$ in actual water samples using NS-CQDs.

| Sample | Standard added (μM) | Standard detected (μM) | Recovery (%) | RSD (%) |
|---|---|---|---|---|
| Lake water | 50 | 50.6 | 101.2 | 2.20 |
| River water | 50 | 49.1 | 98.2 | 1.47 |
| Tap water | 50 | 51.7 | 103.4 | 3.48 |

**Table S4** Detection of HQ in actual water samples using NS-CQDs+$Fe^{3+}$.

| Sample | Standard added (μM) | Standard detected (μM) | Recovery (%) | RSD (%) |
|---|---|---|---|---|
| Lake water | 100 | 103.8 | 103.8 | 2.68 |
| River water | 100 | 101.1 | 101.1 | 1.98 |
| Tap water | 100 | 99.5 | 99.5 | 2.36 |
